# Transcriptional regulators of redox balance and other homeostatic processes with the potential to alter neurodegenerative disease trajectory

**DOI:** 10.1042/BST20170013

**Published:** 2017-11-17

**Authors:** Scott W. Burnside, Giles E. Hardingham

**Affiliations:** 1 Edinburgh Medical School, University of Edinburgh, Edinburgh EH16 4TJ, U.K.; 2 UK Dementia Research Institute at The University of Edinburgh, Edinburgh EH16 4TJ, U.K.

**Keywords:** antioxidants, neurodegeneration, oxidative stress, transcription factors

## Abstract

Diverse neurodegenerative diseases share some common aspects to their pathology, with many showing evidence of disruption to the brain's numerous homeostatic processes. As such, imbalanced inflammatory status, glutamate dyshomeostasis, hypometabolism and oxidative stress are implicated in many disorders. That these pathological processes can influence each other both up- and downstream makes for a complicated picture, but means that successfully targeting one area may have an effect on others. This targeting requires an understanding of the mechanisms by which homeostasis is maintained during health, in order to uncover strategies to boost homeostasis in disease. A case in point is redox homeostasis, maintained by antioxidant defences co-ordinately regulated by the transcription factor Nrf2, and capable of preventing not only oxidative stress but also inflammation and neuronal loss in neurodegenerative disease models. The emergence of other master regulators of homeostatic processes in the brain controlling inflammation, mitochondrial biogenesis, glutamate uptake and energy metabolism raises the question as to whether they too can be targeted to alter disease trajectory.

## Introduction

Reactive oxygen species (ROS) are oxygen-containing molecules which are primarily formed as a result of incomplete oxygen reduction [1–3]. These substances can be broadly split into two main subgroups: free-radicals, such as superoxide, hydroxyl and nitric oxide, which contain one or more unpaired electrons, and non-radical compounds such as hydrogen peroxide. Although low-level basal ROS generation is essential for various fundamental biological roles, in particular protection against infectious/inflammatory damage as well as cell signalling, an imbalance in ROS production versus opposing antioxidant detoxification can lead to disruptions in cellular redox homeostasis [1–3]. Such disturbances often arise as a function of mitochondrial dysfunction and increased transition metal catalysis [3,4]. An increase in global ROS concentrations promotes the destructive interaction of these species with important cell functional components in a process leading to oxidative stress.

The aggregate effects of oxidative stress are largely determined by the critical interaction of ROS with four key cellular constructs inducing lipid peroxidation, glycoxidation, protein oxidation and specific DNA oxidation events, which, in turn, tend to worsen cellular dysfunction in a vicious cycle of cause and effect [1,3,5]. Within the brain, neurons are particularly vulnerable to oxidative stress, due to their high rates of mitochondrial electron transport, non-regenerative post-mitotic status, weak intrinsic antioxidant defences and high lipid content [1,6]. Indeed, lipid peroxidation is generally one of the earliest features of oxidative stress to occur, commonly initiating a series of harmful downstream chain reactions, inactivating vital protein ion channels and reducing overall membrane integrity, accumulatively resulting in significant cellular apoptosis, neuronal excitotoxicity and neurodegeneration over time [7]. As such, neurons require a complex network of antioxidant enzymes and detoxifying species which can work synergistically to mitigate ROS accumulation and maintain a steady redox environment. This involves intrinsic systems, as well as metabolic support and coupling from other cells in the brain, most notably astrocytes [8]. Despite these systems, however, oxidative stress increases in the brain with age. The accumulation of injury, through decades of poor post-mitotic self-repair and modest central antioxidant defence mechanisms which too deteriorate with time [1], suggests a possible explanation for why even low-level oxidative stress infers a propensity towards general age-related declines in cognitive function. This theory has been consistently backed up by numerous *in vivo* models with antioxidant gene knockout commonly affecting a shortened lifespan [9].

## Oxidative stress and Alzheimer's disease

Oxidative stress and mitochondrial dysfunction have, for some time, been associated with the patho-progression of neurodegenerative diseases, including Alzheimer's disease (AD), the commonest cause of dementia worldwide. AD is an age-related, incurable neurodegenerative disorder, characterised by a progressive decline in cognitive function severely affecting daily functional capacity. Both early-onset familial and late-onset sporadic AD are essentially defined by the same histological hallmarks, that is amyloid-beta (Aβ) plaques, neurofibrillary tangles (NFTs) of hyperphosphorylated-tau protein and a loss of synaptic and eventually neuronal integrity. Mechanistically, the pathological cascade leading to synaptic and neuronal loss is thought to involve loss of homeostasis at multiple levels leading to impaired extracellular glutamate regulation and excitotoxicity, inflammation, bioenergetic and metabolic compromise, proteotoxicity as well as oxidative stress [1,5,7,10–12]. These processes are features of several neurodegenerative disorders and involve interactions between multiple cell types in the brain: the vasculature, macro- and microglia, as well as neurons. The involvement of oxidative hypothesis is supported by numerous post-mortem AD findings, with many showing marked escalations in OS as evidenced by increased 4-HNE carbonyls (protein oxidation), parietal 8-OHDG (DNA damage), F2-isoprostanes/MDA (lipid peroxidation), as well as evidence of hypometabolism and ROS scavenger deficits [1,5,7,10,11].

## Oxidative stress and interactions with other pathological processes

Importantly, oxidative stress can play the role of upstream activator and/or downstream effector of many of these other pathological processes common to neurodegenerative disease. For example, ROS imbalance can promote pro-inflammatory phenotypes in innate and adaptive immune cells, which, in turn, can generate ROS as part of their phenotypic switch [12,13]. Impaired glutamate homeostasis leads to inappropriate activation of NMDA receptors, leading to excessive neuronal Ca^2+^ influx, triggering NO and superoxide production by the Ca^2+^-dependent activation of nNOS and NADPH oxidase, respectively [14]. Mitochondrial dysfunction can lead to excessive ROS production due to electron leakage from the electron transport chain (ETC), while ROS itself can damage the ETC components, further exacerbating the problem [15,16]. More generally, metabolic dysfunction, due to cerebrovascular pathology or reduced glucose uptake capacity, also can lead to oxidative stress due to an inadequate supply of glucose to neurons, ordinarily key for NADPH regeneration through the pentose phosphate pathway [8,17]. In AD, oxidative stress can additionally act as both upstream regulator and downstream effector of APP pathology through influences on APP processing, protein clearance, as well as the downstream signals that oligomeric Aβ trigger [1,5,7,10–12].

## Issues surrounding the therapeutic modulation of antioxidant defences

Despite the attractiveness of the oxidative stress hypothesis for neurodegenerative diseases as a key regulatory node to target, trials with common antioxidants, such as vitamins E and C, in a variety of disorders including AD showed no benefit for patients [5,18]. While this could mean that oxidative stress is not a strong regulator of disease trajectory, there is little evidence that these interventions attained therapeutically relevant levels in the brain or successfully ameliorated disease-associated oxidative stress. Preclinical and clinical studies of other antioxidant molecules with different substrate profiles and pharmacokinetics are ongoing: prominent candidates include the flavonoid apigenin, α-lipoic acid, coenzyme-Q10 and *N*-acetyl cysteine [19]. However, these compounds may suffer conceptually similar issues as earlier vitamin studies.

One issue is the subcellular localisation of the desired increase in antioxidant capacity. Mitochondria are both the site of substantial ROS production and the location of damage. The importance of mitochondrial antioxidant systems is reflected in the fact that mammalian mitochondria possess specialised antioxidant systems, including mitochondrially localised isoforms of superoxide dismutase, glutathione peroxidase and peroxiredoxin [6,20,21]. AD, in particular, is associated with mitochondrial defects, functional analysis of which has been driven by cybrid technology, whereby mitochondria from AD patients (usually platelet-derived) are incorporated into special cell lines (termed ρ0 lines) lacking all mitochondrial DNA [22]. Cybrid studies have revealed AD-associated reduction in cytochrome oxidase activity and expression (critical for efficient oxygen reduction), damaged mitochondrial DNA, and impaired membrane potential and ROS overproduction [22]. Mitochondria from AD patient brains are also deficient in α-ketoglutarate dehydrogenase, a key TCA cycle enzyme which generates ROS and is highly sensitive to inhibition by ROS [23]. Thus, targeting mitochondria to limit damage at the source of production is an attractive strategy. The prototypical mitochondrially targeted antioxidant, MitoQ, utilises the positively charged lipophilic triphenylphosphonium (TPP) ion to target the antioxidant ubiquinone moiety to polarised mitochondrial inner membranes [16]. When oxidised to ubiquinol, it is then reduced back to ubiquinone by ETC complex II. MitoQ protects strongly against lipid peroxidation and reduces overall oxidative stress and pathology in a variety of animal models, including ischaemic–reperfusion injury and Alzheimer's and Parkinson's diseases. *In vitro* analysis of neuronal N2a cells incubated with Aβ peptide indicates that introduction of MitoQ can prevent abnormal peroxiredoxin enzymatic expression and maintain a stable mitochondrial configuration while also increasing neurite growth despite pathological predisposition [24]. Likewise, assimilation into a *Caenorhabditis elegans* AD model was shown to extend lifespan, delay Aβ-induced paralysis and amend mitochondrial depletion of lipid membrane and cytochrome complex I/IV [25]. In humans, a phase II clinical trial in Parkinson's disease failed to show efficacy, though it did show that such TPP-targeted therapeutics could be safe (a potential issue since TPP ions can, in theory, depolarise mitochondria when in excess).

Another issue for all antioxidant therapies is whether any single antioxidant molecule can be in the correct place(s) and the correct time(s) and have the right substrate profile to limit ROS-induced damage and subsequent patho-progression. Mammalian cells contain hundreds of gene products directly or indirectly involved in the reduction of ROS of distinct types, in distinct subcellular or extracellular regions, and yet more involved in the detoxification or reversal of damage. Expecting a single molecule to mimic these defences is perhaps expecting too much.

## The Nrf2 pathway, a conceptually different way to boost antioxidant defences

As an alternative approach, attention has turned to pathways and mechanisms that control the expression of endogenous cytoprotective gene clusters *en masse*. A key master regulator of antioxidant defence and detoxification genes is the transcription factor Nrf2, which acts as a hub onto which several stress-associated signals, including oxidative stress and heavy metal toxicity, converge [26,27]. By default, Nrf2 is held in the cytoplasm and targeted for ubiquitin-mediated proteasomal degradation by its interactor/inhibitor Keap1. However, under conditions of oxidative stress, one or more redox-sensitive cysteine residues on the Nrf2 interaction region of Keap1 become oxidised, interfering with the degradation process. Nrf2 then accumulates in the nucleus where it co-operates with small Maf proteins and activates genes containing an antioxidant response element (ARE) in their promoter. ARE-containing genes include catalase and core components of the glutathione and thioredoxin/peroxiredoxin systems, as well as detoxification enzymes like glutathione-*s*-transferases, and so its activation boosts expression of diverse antioxidant systems, potentially more desirable than relying on a single small-molecule antioxidant. Thus, the Nrf2 pathway acts as an endogenous stress response, activated by insults such as mild ischaemic conditions, and contributing to the resulting neuroprotection [28,29].

Importantly, the Keap1-mediated Nrf2 degradation can also be inhibited by small molecules, which are often electrophilic and act by modifying key Keap1 cysteine residues, particularly cysteine-151 [30]. A range of pharmaceuticals and phytochemicals are able to activate Nrf2-mediated gene expression by this method, reviewed elsewhere, and are protective in several animal models of neurodegenerative disease [26,30]. However, the mechanism of Nrf2-mediated neuroprotection may well be non-cell-autonomous, since forebrain neurons express very little Nrf2 when mature and thus their response to Nrf2 activators is weak or non-existent [31–33]. It is likely that activation of Nrf2 in astrocytes is a key mechanism of neuroprotection, since they support robust Nrf2-mediated responses, and astrocytic Nrf2 is sufficient to promote neuroprotection through the induction of glutathione production, which is then released for breakdown and neuronal uptake and usage [31,33,34].

Of translational relevance, Tecfidera, the pharmacological brand name of the fumaric acid ester dimethyl fumarate (DMF), was approved in 2013 for the treatment of relapsing–remitting multiple sclerosis, following significant therapeutic efficacy in clinical testing on an easily maintained safe oral regimen [35]. Preclinical studies showed that the cytoprotective, antioxidant and anti-inflammatory actions of DMF were via Nrf2-dependent mechanisms [36,37]. Aside from demyelination models, DMF has shown efficacy in animal models of Parkinson's disease [38] and in chronic cerebral hypoperfusion [39]. These disease models may be particularly amenable to Nrf2-activating therapy due to the strong association of oxidative stress in their patho-progression: in Parkinson's disease, ROS production from dysfunctional mitochondria is elevated, and further production as a result of neuroinflammation and dopamine metabolism puts further strain on antioxidant defences [40]. In cerebral hypoperfusion (a rough approximation of small vessel disease, [41]), chronic hypoxia can increase ROS generation from complex III, and further ROS production from inflammation may also contribute [41,42]. The key cell types mediating the effects of DMF are unclear, but nevertheless the fact that DMF dampens pathological pathways common to multiple disorders points to clinical trials of DMF for neurodegenerative diseases being an attractive proposition. DMF is not the only Nrf2 activator being pursued clinically. For example, Omaveloxolone (Reata Pharmaceuticals) is a synthetic oleanane triterpenoid compound and a potent Nrf2 activator in clinical trials for Friedreich's ataxia, and autosomal recessive inherited disorder that causes degeneration of spinal and dorsal root ganglia neurons (particularly sensory ones) as a result of mitochondrial dysfunction and ROS overproduction due to the reduced expression of the mitochondrial protein Frataxin [43,44].

## Beyond Nrf2: exploiting other master regulators of homeostasis?

While targeting antioxidant defences driven by Nrf2 offers potentially huge benefits over conventional antioxidant therapy in combatting neurodegenerative disease, there are some caveats. As noted above, the Nrf2 pathway in forebrain neurons is weak, and neurons use other mechanisms to adapt their antioxidant defences and so its effectiveness may be limited by this. Forebrain neurons retain the ability to dynamically regulate many ARE-containing antioxidant genes, but do so via Nrf2-independent mechanisms [45–48]. Synaptic activity controls the expression of ARE-containing genes *xCT*, *Gclc*, *Gclm*, *Gsr* and *Srxn1* among others via other transcription factors that can be recruited to these genes' promoters, including AP-1 and ATF4 [49,50]. Neuronal activity also down-regulated the thioredoxin inhibitor Txnip, by driving the nuclear export of its regulator FOXO [50,51].

A separate, translational issue is the fact many Nrf2 activators are electrophilic, so there is a danger that excessively reactive compounds at excessive concentrations lead to non-specific modifications leading to an exacerbation, rather than amelioration, of oxidative damage [52]. Moreover, chronic administration of Nrf2 activators will trigger robust Nrf2 responses in peripheral organs where the pathway is highly active. This is of potential concern since somatic mutations in Keap1 and Nrf2, resulting in a constitutively active Nrf2 pathway, have been reported in some cancers and confer high tolerance to anti-cancer drugs [53].

**Figure 1.**
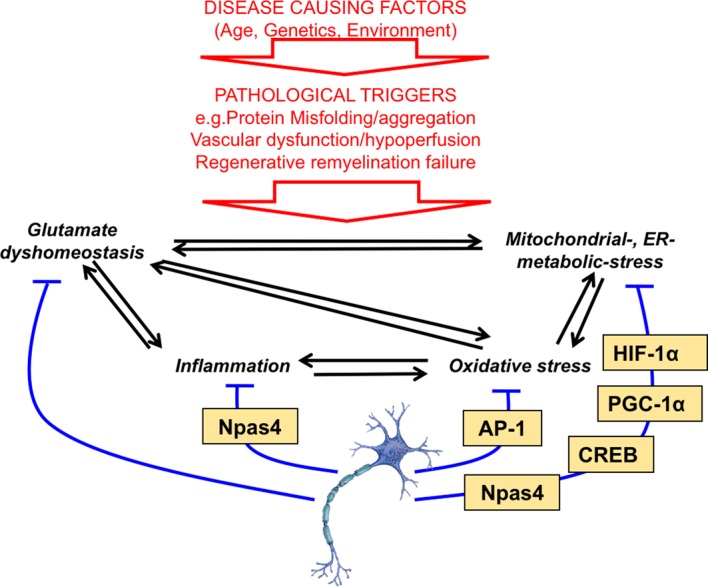
Some transcription factors controlling homeostatic gene expression programmes in neurons. Loss of homeostasis at multiple levels (labelled in black) is a hallmark of a variety of neurological disorders associated with synapse loss, axonal damage or neuronal death. These disorders, which include stroke, traumatic brain injury, Alzheimer's disease, motor neuron disease and the progressive phase of multiple sclerosis, as well as others, have some common features centred on core inter-dependent pathological processes involving loss of glutamate homeostasis and excitotoxicity, inflammation, oxidative stress and metabolic/mitochondrial dysfunction [13,40,55]. These common features contrast with the diverse nature of the pathological triggers (in red). As described in the text, genes whose function is to counter or resolve homeostatic challenges in neurons are under the control of many key transcription factors, labelled here in yellow boxes.

**Figure 2.**
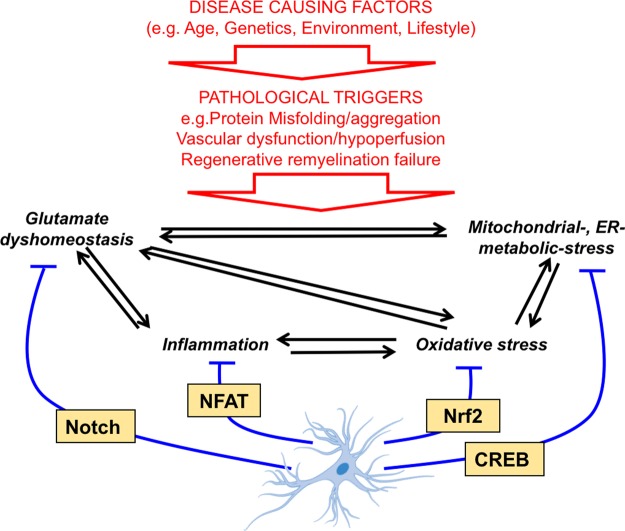
Some transcription factors controlling homeostatic gene expression programs in astrocytes. As described in the text, genes whose function is to counter or resolve homeostatic challenges in astrocytes are under the control of many key transcription factors, labelled here in yellow boxes.

Even setting these issues aside, boosting the brain's intrinsic antioxidant defences may only, at best, offer a partial amelioration of patho-progression, due to incomplete rescue of other homeostatic deficits such as glutamate, metabolic or inflammatory imbalance. These are features of many neurological disorders as well as oxidative stress, and imbalance in one area often leads to imbalance in another area (Figures 1 and 2), and so devising ways of manipulating these processes may offer alternative or complementary ways of altering disease trajectory. Identification of major control nodes of these complex homeostatic processes is the first step towards this. For example, in the context of glucose uptake/metabolism and bioenergetic homeostasis, there is good evidence that this is impaired in the AD brain, through human imaging and PM studies, as well as animal models [54,55]. Hypoxia-inducible factor HIF-1α is a master regulator of both neuroprotective and metabolic genes, including glycolytic pathway genes and glucose transporters, whose expression is reduced in AD, along with HIF-1α itself [54]. Moreover, ectopic HIF-1α is neuroprotective in models of AD [54]. Manipulation of HIF-1α activity can be achieved by inhibiting prolyl hydroxylase domain-containing (PHD) enzymes, which ordinarily target HIF-1α for ubiquitin-mediated degradation. Many classes of PHD inhibitors exist, but several act by chelating iron (PHD is an iron-containing protein) and have a protective effect attributable to HIF-1α stabilisation [54,56].

In neurons, another important regulator of metabolism is the co-activator PGC-1α. PGC-1α is a regulator of the brain's antioxidant defences, neuronal mitochondrial biogenesis [57] and also influences synaptic versus extrasynaptic NMDAR localisation [58] (a key determinant of excitotoxicity). Human post-mortem AD brain tissues have shown significant reductions in PGC-1α mRNA as a function of clinical AD progression [59] and levels are also lower in Huntington's disease, and PGC-1α has been shown to be protective in models of HD and PD [60–65]. PGC-1α is itself regulated by the transcription factor CREB, which controls multiple neurotrophic and neuroprotective genes in neurons, reducing their vulnerability to both excitotoxic and apoptotic insults [66–68]. Npas4 is another activity-responsive transcription factor implicated in promoting homeostasis and neuroprotection in neurons which promotes resistance to apoptotic and excitotoxic insults [69,70]. Npas4 drives the transcriptional repression of the mitochondrial calcium uniporter Mcu, preventing mitochondrial Ca^2+^ overload after chronic NMDA receptor activity [70], as well as the induction of synaptotagmin 10 which mediates resistance to seizure-induced neuronal damage [71]. Neuronal Npas4 also appears to play an immunomodulatory role following trauma, since its knockdown increases astrocyte and microglial activation following stroke in mice [72]. Thus, key transcription factors play key roles in regulating the homeostatic capacity of neurons (Figure 1).

In the context of astrocytes, transcription factors other than Nrf2 are likely to play an important role in distinct aspects of homeostasis (Figure 2). The transcription factor CREB has recently been identified as a master activity-responsive regulator of astrocytic glucose metabolism and lactate export [73] and is neuroprotective when driven in astrocytes [74]. CREB can control many genes in the glycolytic pathway in astrocytes and its constitutive activation up-regulates the capacity of astrocytes to utilise glucose [73]. Since CREB in neurons controls PGC-1α and other neuroprotective genes like *Bdnf* and *Bcl2*, stimulation of CREB activity in the brain may have protective effects at multiple levels acting via multiple cell types [75]. There is emerging evidence that other transcription factors in astrocytes also regulate important homeostatic processes relevant to neurodegenerative disease and are the topic of ongoing investigation. For example, it was recently shown that NFAT in astrocytes is an important regulator of their reactive status, and that astrocyte-specific inhibition of NFAT improved outcomes and reduced plaque load in APP/PS1 mice [76]. Another astrocytic transcriptional programme, regulated by Notch, has been shown to control the expression of glutamate uptake and metabolism genes and functional glutamate uptake capacity [73]. Given that impaired glutamate uptake capacity and transporter expression are associated with ageing and AD [77], the capacity to correct this through altering signalling in astrocytes is an attractive proposition.

## Concluding remarks

The longevity of the human brain is testament to its remarkable capacity for homeostasis. Multiple cell types within the brain are capable of mounting adaptive protective responses to potentially challenging situations in order to maintain the health and function of post-mitotic neuronal circuits. The resolution of homeostatic challenges often occurs on the second scale, involving existing capacity and conduits. However, it is clear that longer term adaptions to homeostatic capacity (arguably a form of allostasis) involving new gene expression also take place. Harnessing the mechanisms that co-ordinate and facilitate brain homeostasis in health may pave the way to maintain this in the face of age or disease-causing agents.

## Abbreviations

4-HNE: 4-hydroxy-2-nonenal; 8-OHDG, 8-Oxo-2′-deoxyguanosine
AD: Alzheimer's disease
ARE: antioxidant response element
Aβ: amyloid-beta
CREB: CaMP response element binding protein
DMF: dimethyl fumarate
ETC: electron transport chain
FOXO: forkhead box O
HIF-1α: hypoxia inducible factor-1 alpha
MDA: 3,4-methylenedioxyamphetamine
NO: nitric oxide
NMDAR: N-methyl D-aspartate receptor
nNOS: neuronal nitric oxide synthase
PGC-1α: peroxisome proliferator-activated receptor gamma coactivator 1-alpha
PHD: prolyl hydroxylase domain-containing
PM: post-mortem
ROS: reactive oxygen species
TPP: triphenylphosphonium
